# Targeting telomerase in myelodysplastic syndrome: imetelstat as a disease-modifying therapy for transfusion-dependent patients

**DOI:** 10.1097/MS9.0000000000003805

**Published:** 2025-09-04

**Authors:** Ahmed Javed, Aimal Alam Khattak, Farman Ullah Khan, Abdul Muhymin Alam Khattak, Bibek Shrestha

**Affiliations:** a Department of Internal Medicine, Ayub Medical College, Abbottabad, Pakistan; b Department of Internal Medicine, Medical Oncology, St. Vincent’s Private Hospital, Dublin, Ireland; c Department of Internal Medicine, Tribhuvan University Institute of Medicine, Kathmandu, Nepal

## Background

Myelodysplastic syndrome (MDS) is a group of disorders characterized by ineffective blood cell production in the bone marrow that can result in decreased levels of healthy blood cells, such as red blood cells (RBCs). According to the Surveillance Epidemiology and End Results study from 2015 to 2019, the number of new MDS cases per 100 000 people each year, adjusted for age differences over 5 years, was 0.2 for those younger than 50, 2.8 in those aged 50–64, and 26.9 in patients aged ≥65[1]. MDS can cause low RBC levels, making patients dependent on frequent blood transfusions. This condition is known as *transfusion-dependent anemia in MDS*. While there are a large number of mutated genes in MDS, these genes are linked to a small number of biological pathways such as signal transduction, transcription factors, DNA damage response, RNA splicing factors, epigenetic regulators, and components of cohesion[2]. Mutations in genes such as SF3B1 (RNA splicing), TP53 (DNA damage response), and TET2/DNMT3A (epigenetic regulation) have been implicated in clonal evolution, genomic instability, and poor prognosis in MDS[2]. Disturbance in these routes lead to dysfunctional hematopoiesis, genomic instability, and clonal evolution. All these are hallmarks of MDS pathogenesis. This editorial article is compliant with the TITAN Guidelines 2025[3].

## Current treatment options

For MDS-related anemia, erythropoiesis-stimulating agents (ESAs) are the current first-line treatment. However, response rates can vary and many patients eventually cease to benefit from these medications due to the decreased sensitivity of erythroid progenitors to ESAs. In patients with low-risk MDS (LR-MDS), a positive response to ESA treatment correlates with endogenous serum erythropoietin levels <500 U/L and minimum RBC transfusion burden (<4 RBC units per 8 weeks). In fact, in studies like EPOANE and the Eastern Cooperative Oncology Group E1996, response rates were found to be higher in patients with endogenous serum erythropoietin concentrations <200 U/L[4]. In LR-MDS patients that are not responsive to ESAs, the available options are lenalidomide (for del5q subtype), hypomethylating agents (HMAs), experimental therapies, or supportive care with chelation therapy[5]. Three drugs have been approved by the US Food and Drug Administration (FDA) for MDS-specific indications: *Decitabine* (another DNA methyltransferase inhibitor) approved in 2006, *Lenalidomide* (a cereblon- and ubiquitin ligase-modulating agent) approved in 2005, and *Azacitidine* (a DNA methyltransferase inhibitor and nucleoside analogue) approved in 2004[6]. The existing data for these drugs are summarized below in Table 1.

**Table 1:** Commonly approved therapeutic agents for myelodysplastic syndromes (MDS), including their class, route of administration, dosing schedules, key clinical trials, and dates of approval

Drug	Class	Mode of administration	Typical dosing duration	Clinical trials	Date of approval
Darbepoetin alfa	Erythropoiesis-stimulating agent (ESA)	Subcutaneous (SC) or intravenous (IV) injection	Every 1–2 weeks	Phase 3 (NCT01362140)	September 17, 2001
Romiplostim	Thrombopoietin receptor agonist (TPO-RA)	Subcutaneous (SC) injection	Weekly	Phase 2 (NCT01213647)	August 22, 2008
Azacitidine	Hypomethylating agent (HMA)	Subcutaneous (SC) or intravenous (IV) infusion	5–7 days per cycle, repeated every 28 days	Phase 3 (NCT01566695)	May 19, 2004
Lenalidomide	Immunomodulatory drug (IMiD)	Oral (capsules/tablets)	Daily for 21 days of a 28-day cycle	Phase 2 (NCT00065156)	February 22, 2017
Luspatercept	Erythroid maturation agent (EMA)	Subcutaneous (SC) injection	Every 3 weeks	Phase 3 (NCT03682536)	November 8, 2019

## Imetelstat – a new therapeutic option

The US FDA approved *imetelstat* (Rytelo), which is a *telomerase inhibitor*, for the treatment of adults with transfusion-dependent anemia and MDS, requiring four or more units of RBCs in an 8-week period, and not responding to, losing response to, or ineligible for ESAs[7]. It is a 13-unit oligonucleotide conjugated to lipids for improving its delivery. Notably, it has been shown to bind *RNA template of human telomerase* and inhibit its catalytic activity[7]. Imetelstat inhibits telomerase activity that results in telomere shortening which eventually causes senescence or apoptosis of malignant hematopoietic cells.

## Clinical studies

The efficacy of imetelstat (Rytelo) was evaluated in the IMerge trial (NCT02598661), which is a multicenter study involving 178 patients with low- or intermediate-1-risk MDS according to the International Prognostic Scoring System. All participants had transfusion-dependent anemia and required at least four units of RBCs over an 8-week period prior to randomization. Eligible patients had either failed to respond to or were ineligible for ESAs and met specific hematologic criteria (absolute neutrophil count ≥1.5 × 10^9^/L and platelets ≥75 × 10^9^/L). Patients with certain genetic abnormalities or prior exposure to lenalidomide or HMAs were excluded[8]. The study primarily aimed to evaluate the rate of transfusion independence, a key indicator of clinical benefit. Imetelstat, a telomerase inhibitor, significantly improved RBC transfusion independence in the IMerge Phase 3 trial (NCT02598661) for non-del(5q) lower-risk MDS patients. The transfusion independence rates were 39.8% for ≥8 weeks, 28.0% for ≥24 weeks, and 17.8% for ≥1 year, compared to 15.0%, 3.3%, and 1.7% with placebo, respectively[9]. These results highlight the potential of imetelstat as a disease-modifying therapy in lower-risk MDS, particularly in patients unresponsive to conventional treatments.

Clinicians should use imetelstat in patients with non-del(5q) lower-risk MDS requiring ≥4 RBC units over a period of 8 weeks who have failed, are ineligible for, or have lost response to ESA based on current evidence. RYTELO is indicated for the treatment of adult patients with low- or intermediate-1 risk MDS with transfusion-dependent anemia requiring four or more RBC units over an 8-week time frame who have not responded to or have lost response to or are ineligible for ESA[7]. Selection should also consider adequate baseline hematologic parameters (ANC ≥1.5 × 10^9^/L and platelets ≥75 × 10^9^/L) and overall clinical fitness.

## Dosage and side effects

Imetelstat is given in an intravenous infusion of 7.1 mg/kg over 2-hour period every 4 weeks[7]. The most clinically relevant adverse events are dose-dependent cytopenias (e.g. neutropenia, thrombocytopenia) and infusion-related reactions. Such toxicities may necessitate dose reductions; growth factor support or temporary treatment holds to maintain patient safety.

Whereas cytopenias as early toxicities are well known, long-term telomerase inhibition might theoretically be associated with impaired regeneration of normal hematopoietic stem cells or other tissue renewal systems. Thus, patients should be followed for bone marrow suppression, secondary malignancies, and organ dysfunction as part of the long-term surveillance.

## Insights and therapeutic implications

The introduction of imetelstat as the first FDA-approved telomerase inhibitor for transfusion-dependent MDS is not just a therapeutic milestone, it represents telomere biology as the connector between targeted therapy and tumor immunology. Central to this view is the immunogenic cascade resulting from telomerase inhibition: critically short telomeres trigger DNA damage responses that allow for the release of Telomeric repeat–containing RNA and chromatin fragments. These nucleic acids activate cyclic GMP-AMP synthase, followed by downstream STING pathway signaling, producing Type-I interferons that facilitate dendritic cell recruitment and enhance tumor antigen presentation[10]. Simultaneously, senescent malignant cells initiate their own cytokine secretory release of pro-inflammatory cytokines (IL-6, TNF-α) through the senescence-associated secretory phenotype, changing the bone marrow microenvironment from immunocompromised to immunoreactive[11]. This dual mechanism of antigen exposure and inflammatory reprogramming creates a “cold-to-hot” transformation in the MDS clones, ultimately allowing for immunity against previously tolerated malignancies.

The convergence with cancer immunotherapy is mechanistically and strategically based. Imetelstat confers telomere dysfunction, and activate stress ligands (e.g. MIC-A/B) which activate NK cells and associated anti-leukemic activity through NKG2D receptors[12], while simultaneously down-regulating immune checkpoint molecules on surviving blasts. This makes combination strategies more attractive: (1) Lenalidomide could enhance CRBN-mediated IKZF1 degradation to enhance killing of clones reduced from telomerase, or (2) hypomethylating agents would finally dedifferentiate CT (cancer-testis) antigens in senescent cells, expanding the antigenic repertoire. This strategy is consistent with current trends in the treatment of cancer. Just as giving immunotherapy before surgery (neoadjuvant) is more effective than giving it after surgery (adjuvant) because immune suppression is eliminated much earlier, giving imetelstat BEFORE other drugs may provide a greater challenge to immune activation – an idea supported by large cancer data analyses[10]. Of significant interest is that immuno-oncology has been shown to achieve a greater tumor-specific T-cell activity and responses from preoperative interval immunotherapy than from postoperative immunotherapy, which offers a conceptual framework that could be applied to MDS by sequencing imetelstat prior to immunomodulators.

Important knowledge gaps create exciting research opportunities. First, as part of the biomarker studies, we want to explore how the immune activation occurs in phases throughout the duration of imetelstat treatment. Second, we want to determine the influence of common mutations, as it appears that imetelstat response may be better in patients with SF3B1 mutations, but baseline immune microenvironments suggest a combination strategy may be warranted[9]. Third, we must determine the best therapeutic sequence, including whether telomerase inhibition should occur first, as this might prime an immune response capable of being tested, similar to therapy regimens in neoadjuvant settings in trials[10]. Translationally, safety and toxicities remain at the forefront, especially when considering that cytopenias caused by imetelstat will likely be worsened with concurrent immunotherapy, which can have limitations defined in protocols.

## Conclusion

The FDA approval of imetelstat for the treatment of patients with low to intermediate-1 risk MDS who are transfusion-dependent and resistant to treatment with ESAs marks an important milestone in the area. Not only does it reduce RBC transfusion dependence, but it may represent a potential disease-modifying strategy by targeting telomerase. In clinical trials, its efficacy has signaled potential as a treatment alternative to conventional therapies.

## Recommendation

It suggests there could be a way forward for imetelstat in the MDS setting. However, the long-term safety data, durability of response, and combination strategies are needed to be investigated to maximize patient outcomes. It is important to make sure it accessible to all patient and efforts should be made on reducing it cost to ensure its widespread availability. Doctors should increase awareness about this medication and encourage it when appropriate. Post-market evaluations are essential to assess its performance and impact once it is in wider use.

In lower-resource countries where cost and access are barriers, partnerships with government and NGOs may be considered to reduce cost of treatment. Inclusion of imetelstat in national essential medicine lists and the implementation of tiered pricing models with manufacturers would improve availability. If necessary local production under voluntary license should also be taken into account to bring down prices.

## Future directions

Future clinical trials may investigate imetelstat in combination with HMAs or with lenalidomide, particularly in patients with overlapping mutations and/or suboptimal response to monotherapy, where the mechanism of action could be attributed to synergistic mechanisms. The synergistic rationale is based on the observation that telomerase inhibition via imetelstat creates telomere shortening and DNA damage, which may enhance the cytotoxic effects of HMAs based on activation of DNA damage response pathways. At the same time, lenalidomide, an immunomodulatory agent, may also facilitate the immune-mediated elimination of telomerase-inhibited clonal cells by activating T-cells and demonstrating anti-angiogenic effects[6]. Telomerase inhibition may sensitize malignant clones to DNA damage from HMAs, while lenalidomide may help stimulate immune-mediated clearance of clonal cells.

## Data Availability

Not applicable.

## Research registration unique identifying number (UIN)

NCT02598661.
